# Field dispersion in uniformly‐excited radial parallel plate waveguides for a compact proton accelerator design

**DOI:** 10.1002/mp.17868

**Published:** 2025-05-12

**Authors:** Morgan J. Maher, Christopher M. Lund, Julien Bancheri, David G. Cooke, Jan Seuntjens

**Affiliations:** ^1^ Medical Physics Unit McGill University Montréal Québec Canada; ^2^ Department of Physics McGill University Montréal Québec Canada; ^3^ Princess Margaret Cancer Centre University Health Network Toronto Ontario Canada; ^4^ Department of Medical Biophysics University of Toronto Toronto Ontario Canada

**Keywords:** dielectric wall accelerator, proton therapy, radial waveguide

## Abstract

**Background:**

Proton therapy (PT) is a beneficial modality for treating certain cancers but remains under utilized due in part to the high cost of existing PT devices. Dielectric wall accelerators (DWAs) are a proposed class of coreless induction accelerators that may present a suitable option for compact and affordable PT. To realize a compact device, acceleration modules must be designed to achieve field strengths approaching 100 MV/m delivered as pulses on the order of nanoseconds.

**Purpose:**

Here, we examine pulse injection into radial parallel plate waveguides as a means of producing high‐intensity, pulsed accelerating fields. We present an approach for understanding the impact of waveguide properties on electromagnetic dispersion as well as a means of accounting for this dispersion to produce suitable accelerating fields.

**Methods:**

Geometric and material properties for a set of waveguides were identified based on existing literature and commonly available materials. An analytic model is presented to describe how waveguide geometry and material affect electromagnetic dispersion in a waveguide. Simulations performed in COMSOL Multiphysics are used to calculate a transfer function for the set of waveguides, which provide a means of determining the waveguides output for arbitrary inputs and vice versa. The simulation results are compared to the analytic solution and used to explore alternate matching conditions at the beampipe of the accelerator.

**Results:**

Overall, radial waveguides provide a passive enhancement of the injected pulse, with enhancement of high‐frequency components found to be proportional to the square root of the ratio of outer radius to inner radius of the waveguide. Dispersion in the waveguide caused by the radial propagation of the pulse depends on multiple waveguide properties (outer radius, inner radius, material) and leads to reduced enhancement at lower frequencies. The field enhancement in the waveguides reduces the peak voltage required to achieve the desired accelerating field strength. However, dispersion alters the temporal profile of the applied pulse, resulting in a distorted field at the inner radius. Using the transfer function, it is possible to determine the shape of the pulse required to achieve a suitable accelerating field for a given waveguide design.

**Conclusions:**

Passive field enhancement occurred in all waveguides and across all frequencies studied in this work. As such, radial parallel plate waveguides could help to reduce the high voltages required from upstream switching networks. The analytic model can be used to select waveguide parameters that provide a suitable enhancement of the upstream voltage pulse to achieve the high field strengths required for a compact accelerator. However, pulse dispersion must be accounted for. If upstream pulse shaping can be achieved to account for electromagnetic dispersion in the waveguide, pulse injection into radial parallel plate waveguides could be a suitable mechanism for field generation in a DWA.

## 1 INTRODUCTION

Proton therapy (PT) has theoretical advantages over conventional photon radiotherapy due to the depth‐dose profile of protons. The Bragg peak, a high‐dose region near the end of the protons' range, results in potential escalation of tumor doses and/or minimization of healthy tissue doses relative to photon radiotherapy.^1^, ^2^ However, PT remains under utilized, with the upfront cost and the size of PT equipment cited as key reasons for a lack of clinical uptake.^3^, ^4^ The proton accelerator is one of the biggest, heaviest, and most expensive pieces of clinical PT devices.^3^, ^4^

**FIGURE 1:**
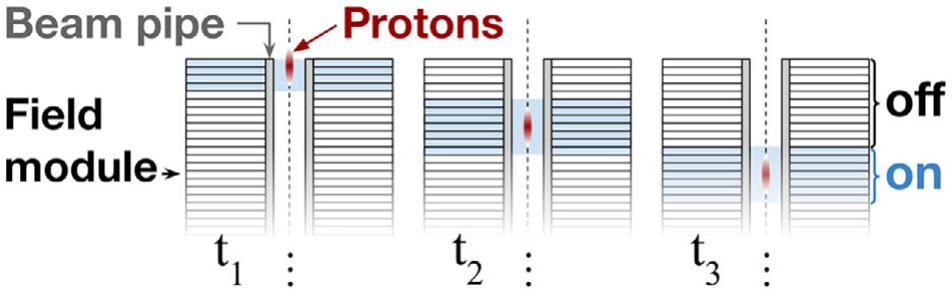
Simplified schematic of DWA operation. As a proton bunch enters the beam pipe, field modules stacked along the beam axis are sequentially switched on in coordination with the protons' trajectory. A cross‐section of the device is shown at three time points (indicated by $t_{n}$) to illustrate the relation between proton bunch location and active field modules. DWA, Dielectric wall accelerators.

Dielectric wall accelerators (DWAs) are a proposed class of coreless linear induction particle accelerator that may provide a more compact, lightweight, and affordable architecture relative to existing clinical proton accelerators. Particle acceleration in DWAs is achieved using short‐lived, high‐intensity electric fields applied at the wall of a dielectric beam pipe. By sequentially delivering these electric field pulses along the length of the beam pipe, a virtual traveling wave is created, which can be temporally coordinated with the particle bunch trajectory (Figure 1). This ensures that the particle bunch experiences continuous acceleration while each physical region of the accelerator is only subjected to the field for the duration of the field pulses. The short duration of the electric field pulses reduces the duty cycle (and therefore power consumption) as well as the overall electrical stress on the system. The latter is an important consideration when striving for a compact accelerator design, since electromagnetic breakdown limits the maximum accelerating field that can be achieved. Empirical evidence suggests that breakdown thresholds increase with decreasing pulse duration. Although the exact relation depends on many design factors, scaling of the breakdown threshold with slopes ranging from $t_{p}^{-1/6}$ to $t_{p}^{-1/3}$ have been suggested for pulses of width $t_{p}$,^5^ while a similar relation for RF fields indicates a scaling of $f^{1/3}$ for fields of frequency f.^6^ Existing research into HGIs, which are constructed of alternating layers of conductors and insulators, have indicated that nanosecond pulses could allow for fields of up to 100 MV/m.^5^, ^7^, ^8^, ^9^ If a beam pipe can be constructed using a high‐gradient insulator and these fields can be realized in a DWA, clinical treatment energies of 70 to 250 MeV could be produced in an accelerator of only a few meters long. Overall, the DWA's linear design (lack of bending magnets) and high field strengths enabled by nanosecond fields promise a compact and affordable device.

Although research into similar concepts dates back to the 1960s, the modern form of the DWA, along with its potential applications to medical physics, was first proposed and developed by researchers at Lawrence Livermore National Laboratory.^10^ They undertook significant development efforts, including overall design proposals as well as the testing of key components, such as switches, high‐gradient insulators (HGIs), and other critical devices. A small‐scale prototype (3 cm) of an operational DWA segment was built as a proof of concept, with plans for a full‐scale device.^11^ However, the realization of a full‐scale prototype was never achieved due to technical and logistical challenges. At the time, some of the key issues highlighted in the literature included switch failure and parasitic coupling, which hindered the scalability of the device. Although their group did not pursue further work, evidence suggests that the concept is still feasible.^12^ Therefore, our group is working to reassess challenges in constructing a medical DWA. Under the title of ‘Project IMPACT’, we aim to take a multifaceted approach that includes working to overcome technical limitations, developing models to assess and optimize device performance, and evaluating the integration of a DWA into the clinical landscape.^13^, ^14^, ^15^ This paper focuses specifically on developing a model to assess and optimize a new approach at generating the accelerating fields that uses alternate switching mechanisms and incorporates geometries that mitigate parasitic coupling.

To generate accelerating fields, DWAs employ a method similar to conventional linear induction accelerators, whereby a field‐generating module produces a potential difference across a gap between two conductors positioned at the wall of the beam pipe. By placing many of these field‐producing gaps along the beam axis, the particle bunches experience continuous acceleration. However, the field production in conventional induction accelerators relies on bulky magnetic cores, and thus large modules, that force an increased spacing between the gaps and reduce the effective acceleration gradient of the accelerator. DWAs, on the other hand, avoid the use of magnetic cores (early development of similar accelerators can be found under the name “coreless” or “ironless” induction accelerators^16^, ^17^) and instead employ thin parallel plate structures that can be placed closely together as shown in Figure 1. Recent prototypes have employed pulse‐forming networks (PFNs), but this design approach suffers from drawbacks. First, PFNs generally require long charging times relative to the duration of the pulse that is produced. This subjects components to significant electrical stress and has lead to switch failure.^18^, ^19^ The long charging times also limit the operation frequency of the device, which could have implications on treatment times or on the potential use of a DWA for time‐sensitive treatments such as FLASH radiotherapy. Additionally, the pulse‐generating mechanism in PFNs, which relies on the discharge of transmission lines, produces flat top field pulses. These flat top pulses do not generate a focusing force along the direction particle of motion, which can affect the energy spread of the bunch and stable beam transport.^14^ An alternative to PFNs – based on pioneering work at SLAC^20^, ^21^ – involves the injection of pre‐formed pulses directly into a parallel plate structure. Building on this, our group has conducted studies of diode‐based pulsers which can produce high‐voltage nanosecond‐scale pulses at repetition rates that could be suitable for use in a DWA.^13^ This approach also opens up the potential for pulse shaping, which we have shown can be implemented to maintain stable beam transport.^14^ Provided a suitable method for delivering these pulses to the beam pipe can be identified, this approach could help bypass the switch failures observed in past designs while offering advantages in beam stability and repetition frequency.

Although various geometric configurations have been studied, to our knowledge only rectangular parallel plate structures have been considered and implemented in prototyping for field delivery in a DWA^18^, ^20^, ^21^, ^22^, ^23^ However, the use of geometries with radial symmetry about the beam pipe has been discussed with interest in the literature^8^, ^22^, ^24^ due to their potential advantages over other designs. Specifically, radial geometries avoid magnetic (parasitic) coupling between adjacent field‐generating sections because, unlike strip geometries where magnetic field lines close through adjacent modules, the magnetic fields in radial waveguides close in the same plane as the waveguide itself.^8^ Additionally, assuming equal voltage between the plates, radial geometries produce a higher field since the field is supplied uniformly around the entire beam pipe.^8^ However, the radial parallel plate structures employed in these designs have a characteristic impedance (Z) that varies radially as:

(1) $$\begin{array}{c} Z\left(r\right)=\sqrt{\frac{\mu }{\varepsilon }}\frac{d}{2\pi r}, \end{array}$$

where μ is the permeability of the dielectric, ε is the permittivity of the dielectric, and d is the plate separation.^25^ This radial variation in impedance leads to pulse distortion.^26^ Additionally, the inverse dependence on radius results in low impedance for large radii, which can require a large current to achieve a given voltage.^8^ Attempts to avoid pulse distortion have focused largely on the design of uniform impedance radial structures, which can be achieved by radial variation of the permittivity, permeability or plate separation.^26^ Radial variation of the plate separation, can be found in the literature on the original development of coreless induction accelerators (for example, the RADLAC accelerator.^27^) Radial variation of the relative permittivity (ε) was also studied by Nelson et al.^24^ Although these approaches resolve the problem of pulse distortion, they introduce challenges in practice, particularly in the context of a compact and affordable device. Varying the plate separation requires increasing the plate separation linearly (see Equation 1) as a function of radius. This in turn increases the spacing of the accelerating gaps along the beam axis, thus reducing the net gradient (or requiring a higher voltage to maintain the desired gradient) at the beam pipe and impacting the compactness of the DWA. Indeed, the coreless induction accelerators that employ this solution were not designed in the context of creating a compact and affordable medical device and therefore faced significantly loosened constraints on size and field strength relative to a DWA. The primary efforts in designing uniform impedance radial structures for a DWA instead focused on the variation of the relative permittivity, where Nelson et al.^24^ radially varied $\varepsilon_{r}$ from 1.9 to 40 for a structure of outer radius 14 cm. This approach would likely create challenges in manufacturing (sourcing materials with high relative permittivity, ensuring radial variation is consistent in each field‐generating section) which could increase the cost of the accelerator as a result. Additionally, an outer radius larger than 14 cm may be necessary, thus requiring an even more dramatic range of relative permittivities. Ultimately, a suitable approach for handling pulse distortion is still needed in order to fully capitalize on the benefits that radial geometries offer for a DWA. Despite the potential benefits of a DWA‐based medical device, to our knowledge, no DWA has yet been constructed beyond initial prototyping due to challenges associated with the accelerator design.

In this work, we examine an alternative approach to electric field generation involving external pulse injection into radial parallel plate waveguides. Specifically, we apply electromagnetic theory and numerical modeling in an attempt to understand and characterize how the geometric and material properties of radial waveguides impact electromagnetic dispersion. Furthermore, we use this information as a means of relating the input pulse (which has implications for the circuit design) to the output field (which will affect the particle dynamics as they traverse the device). This work presents a critical step in assessing the feasibility of our novel approach to electric field generation for a DWA, which combines pulse injection and radial lines, as a means of bypassing some of the challenges faced in the original development. Additionally, the numerical approach presented here has been developed in such a way as to be generalizable to more complex geometries, providing an important foundation for future development and optimization work. The basic understanding of the operation of radial waveguides presented in this paper underpins any further ongoing work on circuitry integration, impedance considerations, and power‐focusing requirements to bring the appropriate accelerating field to the particle bunch.

## 2 METHODS

### 2.1 Waveguide characteristics

The radial parallel plate waveguides studied analytically and numerically in this work were characterized by a set of geometric and material parameters. Geometric parameters included the inner radius ($r_{\text{in}}$), outer radius ($r_{\text{out}}$), and the plate separation (d) of the structure. The insulating material within the waveguide was defined using the relative permittivity ($\varepsilon_{r}$), relative permeability ($\mu_{r}$), and electrical conductivity ($\sigma_{\text{el}}$; numerical studies only). The parallel plates were assumed to act as perfect electrical conductors. The parameter space for geometric and material parameters was selected based on existing literature and on physical constraints of the system.

Inner radii were selected to reflect a range of possible beam pipe radii (0.5  to 2.5 cm in 0.5 cm increments). Outer radii ranged from 10 to 50 cm in 10 cm increments. Plate separations of 0.1 , 1 , and 10 mm were studied, where 1 mm is a value studied previously in the literature,^22^ while the others allow for a study of potential impacts across multiple orders of magnitude that may be feasible with readily‐available materials.

The relative permittivity was examined for vacuum ($\varepsilon_{r}=1$), a dielectric with material properties in the range of values found for commercially available printed circuit board (PCB) material ($\varepsilon_{r}=3.4$), and silicon ($\varepsilon_{r}=11.7$). The relative permeability was unity ($\mu_{r}=1$) for all studies as only non‐magnetic materials were of interest. For the simulation work, electrical conductivity is a required material parameter. The electrical conductivity was set to a low value ($\sigma_{\text{el}}=1\times 10^{-12}$ S/m) for all numerical studies to emulate a realistic insulator with minimal conductivity.

### 2.2 Analytic enhancement factor

The analytic enhancement factor (A) provides a theoretical prediction for the dispersion of electromagnetic waves propagating radially inwards in an axially symmetric parallel plate waveguide. Maxwell's equations and the boundary conditions imposed by the conductive parallel plates were used to derive expressions for the electromagnetic fields in the cylindrically symmetric waveguide. To satisfy the conditions for particle acceleration, the electric field used for acceleration must align with the direction particle propagation.^28^ Keeping with conventional notation for cylindrical coordinates, the particles propagate along the z‐direction. To achieve particle acceleration along z, the electric field produced at the wall of the beampipe–and therefore within the waveguides–must also be oriented along the z‐direction. In radial parallel plate waveguides, the transverse electromagnetic (TEM) mode is the dominant mode that satisfies this condition. It has no cutoff frequency, and uniform excitations, which have no variation in the field along the θ‐ or z‐directions, provide the appropriate initial conditions to couple into this mode. The electric field for the TEM mode is governed by the expression:

(2) $$\begin{array}{c} E_{z}\left(s,\omega \right)=\frac{\beta_{s}}{i\omega \varepsilon }\left[\kappa_{1}J_{0}\left(\beta_{s}\;s\right)+\kappa_{2}Y_{0}\left(\beta_{s}\;s\right)\right], \end{array}$$

where s is the radial distance from the origin, ω is the angular frequency of the field, $\beta_{s}=\sqrt{\omega^{2}\varepsilon \mu }$ is the wave number in the radial direction, $\kappa_{1}$ and $\kappa_{2}$ are constants determined by the initial conditions of the system, and $J_{0}$ and $Y_{0}$ are, respectively, zeroth‐order Bessel functions of the first and second kind. The moduli of the Bessel functions of the first and second kind can both be expressed as $\sqrt{J_{0}^{2}+Y_{0}^{2}}$,[^29^ section 10.18], such that the magnitude of the field is represented by:

(3) $$\begin{array}{c} |E_{z}\left(s,\omega \right)|=\left|\frac{\beta_{s}}{\omega \varepsilon }\right|\sqrt{\kappa_{1}^{2}+\kappa_{2}^{2}}\sqrt{J_{0}{\left(\beta_{s}\;s\right)}^{2}+Y_{0}{\left(\beta_{s}\;s\right)}^{2}}. \end{array}$$

To further simplify the expression, $\sqrt{J_{0}^{2}+Y_{0}^{2}}$ can be replaced by the magnitude of the zeroth‐order Hankel function ($H_{0}=J_{0}\pm iY_{0}$). By comparing the amplitude of the $E_{z}$ field at the inner radius to that at the outer radius, we obtain a frequency‐dependent expression for the enhancement of the field within the waveguide:

(4) $$\begin{array}{c} A=\frac{|E_{z}\left(s=r_{\text{in}}\right)|}{|E_{z}\left(s=r_{\text{out}}\right)|}=\frac{|H_{0}\left(\beta_{s}\;r_{\text{in}}\right)|}{|H_{0}\left(\beta_{s}\;r_{\text{out}}\right)|}, \end{array}$$

which is independent of the initial conditions (i.e. $\kappa_{1}$ and $\kappa_{2}$).

### 2.3 Numerical frequency response

Transfer functions can be used to model a system's output for any possible input or vice versa. In the context of this work, it is obtained numerically by comparing the frequency spectrum of a reference pulse input as the excitation for a simulated waveguide to the spectrum of the pulse output by the simulation. By approximating the behavior of the waveguide as a linear system, the output pulse (h) is related to the input pulse (f) through the convolution of the input with a waveguide‐specific function (g) as:

(5) $$\begin{array}{c} h=f*g. \end{array}$$

A deconvolution can be performed in the frequency domain to extract the waveguide‐specific transfer function (G):

(6) $$\begin{array}{c} G=\frac{H}{F}, \end{array}$$

Once the transfer function has been calculated for a given waveguide based on the reference excitation and simulation output, it can be used to transform between arbitrary input and output pulses without needing to repeat the simulation. For example, for a known arbitrary input pulse $f_{a}$, the output pulse $h_{a}$ can be calculated as:

(7) $$\begin{array}{c} h_{a}=\mathrm{ifft}\left(G\cdot \mathrm{fft}\left(f_{a}\right)\right) \end{array}$$

Conversely, and importantly for this work, the input required to produce an arbitrary output pulse can be calculated:

(8) $$\begin{array}{c} f_{a}=\mathrm{ifft}\left(\frac{\mathrm{fft}(h_{a})}{G}\right). \end{array}$$

This means that one can calculate the required input into a waveguide in order to achieve a desirable accelerating field.

**FIGURE 2:**
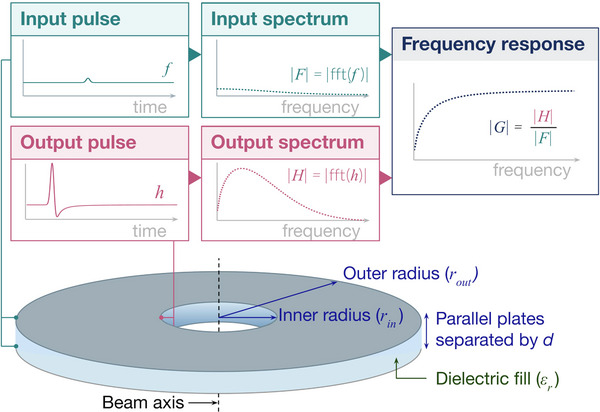
Methods for determining numerical frequency response. Simulated waveguides with defined geometric (inner radius, outer radius, and plate separation) and material (dielectric fill) parameters are excited with a Gaussian input pulse at the outer radius. The output of the waveguide is measured at the inner radius, and the spectra of the two pulses are used to calculate the frequency response.

Additionally, the magnitude of the transfer function (|G|), which we will define as the numerical frequency response in this work, can be used as a metric for comparison between theory and simulation. Since both the frequency response and the analytic enhancement factor described in Section 2.2 relate to the frequency‐dependent enhancement incurred by the waveguide, they provide an intuitive metric for understanding how the system affects a given excitation. A schematic describing the approach for calculating the frequency response using simulated waveguides can be seen in Figure 2. Although the figure focuses specifically on the frequency response, the full transfer function (G) is obtained through the same process by using the full complex‐valued input and output spectra rather than the magnitude alone.

### 2.4 Simulations

An axially symmetric model of radial parallel plate waveguides was built in COMSOL Multiphysics (Version 5.5). The transient electromagnetic waves (TEMW) physics interface within the Radio Frequency (RF) module was used to perform time‐dependent studies. These study packages implement the finite element method to solve Maxwell's Equations in their differential form, and are commonly applied to problems involving the propagation of electromagnetic pulses.

The boundaries of the simulation space are defined by the plates of the waveguide and the inner and outer radii. The plates were assigned as perfect electrical conductors. The boundaries at the inner and outer radii were assigned as voltage‐driven user‐defined lumped ports. The lumped port at the outer radius was used as the excitation point and was assigned an impedance matched to the impedance of the radial waveguide, as defined in Equation 1. The lumped port at the inner radius was used to measure the outgoing waveform and was studied for two conditions: (1) matched impedance, representing full transmission of the field into the beam pipe and transfer of energy to the beam, and (2) infinite impedance, representing an open boundary condition where a complete reflection occurs at the beam pipe and no energy is transferred to the beam.

To improve simulation efficiency and ensure adequate resolution of the signals, a custom mesh was implemented. A rectangular mesh was swept along the radial direction (direction of wave propagation), where the radial length of each element was defined as one tenth the minimum wavelength of the reference excitation. Along the *z*‐direction, the maximum element size was similarly constrained, with the added requirement that at least 5 mesh‐elements be present across the thickness of the plate.

The purpose of the reference excitation is to provide sufficient bandwidth to calculate the frequency response for all frequency components of the desired accelerating field. In this work, a frequency range from 0 to 20 GHz was selected, which provides complete coverage (with a ∼10 GHz buffer zone) for nanosecond‐scale pulses with rise times in the hundreds of picoseconds. A Gaussian pulse with a three‐standard deviation width of 20 GHz was selected as the reference excitation to meet these criteria.

Custom time stepping was implemented to adequately resolve the wave in time and space. Specifically, the following expression was implemented:^30^

$$\begin{array}{c} \Delta t=\frac{C}{Nf_{\max}}, \end{array}$$

where C=0.1 is the Courant number and was identified numerically, N=10 is the number of mesh elements per local wavelength, and $f_{\max}=20$ GHz is the maximum frequency based on the three‐sigma width of the Gaussian reference excitation.

The simulations were terminated at three times the one‐way transit time ($t_{\text{trans}}$) of the given waveguide, where the transit time was defined as the time required for a wave traveling at velocity v=c/n (where c is the speed of light in vacuum, and $n=\sqrt{\varepsilon_{r}\mu_{r}}$ is the refractive index) to propagate from the outer to the inner radius. This choice was made to avoid including secondary reflections of the pulse in the analysis, which can occur for $t>3t_{\text{trans}}$. Although the pulses used to obtain the transfer function are short relative to the transit time of the waveguides studied, it is important to note that the overlap of the primary pulse with secondary reflections could become a concern for longer pulses. Limiting the pulse width to less than $2t_{trans}$ ensures that the trailing edge of the primary pulse does not overlap with the leading edge of a secondary reflection. In addition to restricting the pulse width, this constraint can be satisfied by modifying the waveguide design. Specifically, adjusting the material properties to influence wave velocity or altering the geometry to affect the propagation distance can be used to modify $t_{trans}$.

In analyzing the results and obtaining the frequency response as described in Equation 6, a window function was applied to the output signal prior to converting in into the frequency domain in order to minimize spectral leakage. A Tukey window (commonly used for windowing transient signals) of length $3t_{trans}$ was selected to match the length of the signal, and the taper length was maximized in such a way as to minimize leakage while ensuring that the full pulse is contained within a region where the window has a value of 1. Specifically, a cosine fraction of α=0.66 was selected, which means that the first and last third of the window correspond to cosine tapers, while the central third is flat with a value of 1. The time for the pulse to propagate from the outside in corresponds to $t_{trans}$, therefore the pulse arrives at the inner radius within the central third of the window. Frequencies below $1/t_{trans}$ will be affected by the windowing technique.

### 2.5 Dispersion and pulse forming

Under idealized conditions, the field from the waveguide is transmitted into the beam pipe and its energy is fully transferred to the particle bunch such that no field is reflected back into the waveguide. Both the analytic enhancement factor and the numerical frequency response calculated from simulations with matched impedance (calculated using Equation 1) boundary conditions at the inner radius describe this behavior. The analytic enhancement factor was used to visualize and understand the effect of changing geometric and material parameters on dispersion. This was then compared against the numerical frequency response to assess the performance of the simulations.

In reality, the energy transmitted into the beam pipe won't be fully transferred to the particle bunch, and some of the field will be reflected back into the waveguide. An extreme example occurs when no particle bunch is present and all of the field is reflected back into the waveguide. To describe this scenario, simulations with mismatched infinite impedance boundary conditions at the inner radius were used to determine the numerical frequency response.

To better understand how the dispersion in various waveguide designs influences upstream pulse shaping requirements, the transfer functions of the waveguides were used to calculate the input required to achieve a desired output (see Equation 8). The output was defined as a 1 ns FWHM pulse with a nonzero linear time variation, posited by Lund et al. as a means of maintaining longitudinal control of particle bunches in a DWA.^14^ A linear time slope of 0.1n/s was selected for this work, although the technique can be applied in the same way for arbitrary linear slopes or other desired output pulses.

## 3 RESULTS

### 3.1 Dispersion for matched beam pipe conditions

**FIGURE 3:**
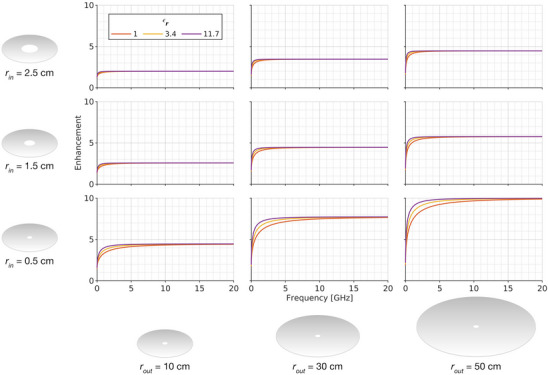
Geometric and material dependence of field enhancement. Each tile on the grid represents a unique waveguide geometry, with distinct inner radii ($r_{\text{in}}$; rows) and outer radii ($r_{\text{out}}$; columns). The frequency‐dependent field enhancement, as calculated using the analytic enhancement factor is plotted in each tile for materials of different relative permittivities ($\varepsilon_{r}$).

The analytic enhancement factor was used to explore how the waveguides’ parameter space (geometry, material) impacts the dispersion the waveguide. Field enhancement (i.e., enhancement >1) was observed for all frequencies of all waveguides studied, which can be seen in Figure 3. A frequency dependence of the enhancement factor was observed, with lower frequencies experiencing less enhancement than higher frequencies. In the upper range of studied frequencies, all waveguides showed saturation of the enhancement. Plate separation was found to have no impact on dispersion. The inner and outer radii impacted the enhancement across all frequencies, with decreasing inner radius and increasing outer radius yielding higher enhancement. The impact of relative permittivity was mainly observed at lower frequencies, with higher relative permittivity yielding more enhancement than lower relative permittivity for a given waveguide geometry. The enhancement factors are visualized for a representative set of waveguide geometries with differing material properties in Figure 3.

**FIGURE 4:**
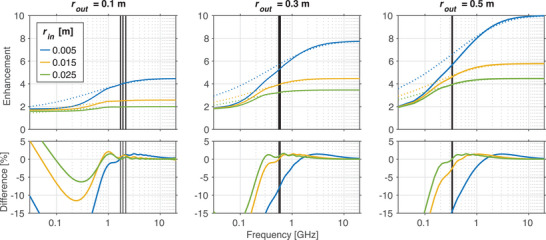
Dispersion of field enhancement. Comparison of numerically calculated frequency response (|G|, solid) and theoretical enhancement factor (A, dotted) for waveguides with a relative permittivity of 3.4 (PCB). Percentage errors between the numerical frequency response and theoretical enhancement factor are plotted in the bottom row. The black vertical lines in each plot correspond to the transit frequency ($1/t_{\text{trans}}$) for increasing $r_{\text{in}}$ values from left to right. PCB, printed circuit board.

The analytic enhancement factor was compared to the numerical frequency response by calculating the percentage difference between the data. The comparison of the analytic enhancement factor and numerical frequency response for the reference waveguide is shown in Figure 4.

### 3.2 Dispersion for mismatched beam pipe conditions

**FIGURE 5:**
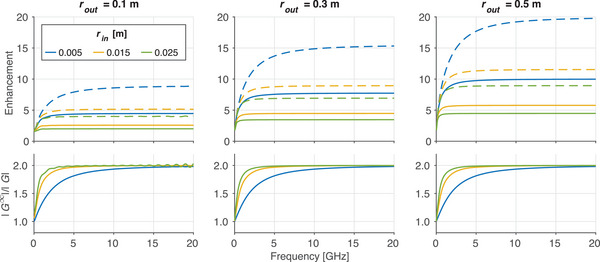
Inner radius impedance conditions. Field enhancement, as calculated using the numerical frequency response, is shown for matched beam pipe boundary conditions (|G|, solid) and infinite‐impedance beam pipe boundary conditions ($|G^{\infty }|$, dashed) for waveguides with a relative permittivity of 3.4 (PCB) with various inner and outer radii. The relative enhancement between the two boundary conditions is plotted in the bottom row for each waveguide geometry. PCB, printed circuit board.

The numerical frequency response was calculated twice for the set of waveguides with differing boundary conditions at the inner radius: (1) with matched impedance, and (2) with infinite impedance. The numerical frequency response is plotted for the reference waveguide in Figure 5.

### 3.3 Pulse forming

**FIGURE 6:**
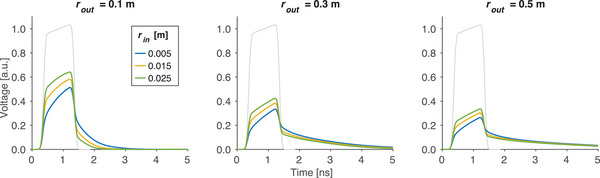
Calculated input excitations. Input excitations calculated using the numerical frequency response and the ideal output waveform for each considered waveguide geometry.

For the specified accelerating field, the numerical frequency response was used to calculate the required input excitations for the set of waveguides. The calculated input pulses for the various waveguide geometries (with $\varepsilon_{r}=3.4$) are compared in Figure 6, with the desired output waveform overlayed in gray. The input pulses were then used to excite a computational waveguide to study the produced output field. The ideal accelerating field used to calculate the input pulse and the simulated output waveform were compared and found to be in agreement, with residuals below 0.1% of the peak voltage.

## 4 DISCUSSION

The analytic enhancement factor provides functional insight into how the waveguide parameters affect electromagnetic dispersion for inward propagating waves in radial parallel plate waveguides. All inward propagating frequencies in the radial waveguide experience some degree of enhancement governed by the ratio of Hankel functions described in Equation 4. The relationship indicates that inner and outer radii are the geometric parameters of interest, since the analytic enhancement factor is independent of plate separation. These results are supported by the numerical data. However, it is important to note that the results presented focus entirely on the TEM mode. As discussed in section 2.2, this assumption arises from our use of uniform excitations, which couple into the TEM mode alone. For alternative excitation mechanisms, where the initial conditions may allow for coupling into higher order modes, the impact of plate separation may need to be reevaluated since the cutoff frequencies for certain modes depend on plate separation.

The electromagnetic properties of the insulating material, namely the relative permittivity and permeability, also affect enhancement. A strong frequency dependence is observed, particularly in the lower frequency regions. The plateau in the enhancement factor that occurs at higher frequencies can be understood by noting that the high‐frequency limit of the enhancement factor can be approximated as:

(9) $$\begin{array}{c} \lim_{\omega \to \infty }A\approx \sqrt{\frac{r_{\text{out}}}{r_{\text{in}}}}. \end{array}$$

This effect can be seen by observing the plateau regions in Figure 3. For example, the enhancement factor in the plateau region is identical for the data along the diagonal from bottom left to top right. Each of these waveguides has a 20:1 outer radius to inner radius ratio, and thus a exhibits a high‐frequency enhancement of $\sqrt{20}\approx 4.47$. The high frequency limit also confirms that the impact of the material properties on field enhancement is present at lower frequencies but diminishes at higher frequencies. Likewise, the value of the inner radius itself affects the rate at which the frequency‐dependent enhancement converges to the high‐frequency limit. Considering the expression for the impedance of a radial parallel plate structure noted in Equation 1, one can arrive at an expression equivalent to the limit shown in Equation 9 by assuming optimal power transfer between the inner and outer radius. Specifically, assuming the power P can be related to the voltage and impedance using the expression $P=V^{2}/Z$, and assuming power conservation between the input and output, the relation between voltage at the outer radius and voltage at the inner radius reduces to $\sqrt{r_{\text{out}}/r_{\text{in}}}$. Through this lens, changes to the voltage pulse can be attributed to two factors: (1) a concentration of the power contained in the pulse as it propagates from the outer radius inwards, which causes an enhancement in the field, and (2) frequency dependent losses due to dispersion incurred within the structure (analogous to the $S_{2,1}$ scattering parameter employed in electrical engineering). Since we aim to understand overall changes to the applied excitation, we focus on the combined impact of these two effects.

Overall, the analytic enhancement factor suggests that field enhancement is primarily impacted by the ratio of the outer and inner radii. Depending on the capabilities of the switching network, the ratio of outer to inner radii can be selected to enhance the pulse such that the desired field strength of 100 MV/m is achieved. This is observed in Figure 6 where waveguides with smaller outer radii and/or larger inner radii require higher input pulse amplitudes in order to achieve the ideal output pulse. The benefit of field enhancement must be balanced against a preference towards smaller outer radii to reduce the size and weight of the accelerator and constraints on the lower bound of the inner radius due to beam physics considerations. Values for inner and outer radii must also be selected to ensure that the pulse produced by the switching network is shorter than twice the transit time of the waveguide. Finally, as has been noted, the values of both inner and outer radii individually influence the dispersion characteristics of the system, further emphasizing the need for careful selection of these parameters.

The numerical frequency response provides a computational means of describing dispersion incurred by a waveguide. Although it does not provide the information on the functional dependence between the waveguide parameters and the voltage enhancement offered by the analytic approach, it has the added benefit of allowing for the simulation of increasingly complex systems that may be difficult or impractical to describe using electromagnetic theory alone. The comparison between analytic and numerical approaches is depicted in Figure 4. Deviations below the transit frequency, defined as $1/t_{\text{trans}}$, are expected since the period of the waves start to exceed the transit time of the waveguide, resulting in poor sampling of low frequency components in the output pulse used to calculate the numeric frequency response. For frequencies exceeding the transit frequency of the waveguide, agreement between simulation and theory was within 3% for all waveguides except those with inner radii of 0.005 m. In this case, the discrepancy arises from the simplifying assumptions in our analytic solution. Sub‐wavelength holes are known to introduce complex effects that become difficult to describe analytically.^31^, ^32^ Since the analytic approach described in Equation 4 considers the TEM mode only, it does not account for complex field behavior that can occur for low frequencies in the vicinity of the inner radius. For larger inner radii, these sub‐wavelength discrepancies occur below the transit frequency. Although the analytic solution holds utility in obtaining a functional description of the impact of waveguide parameters on the field produced at the beam pipe, this result highlights one benefit of EM solvers, which provide a fuller picture of complex behaviors.

The infinite boundary condition was used to test the numerical frequency response beyond the idealized case described analytically, representing waveguide behavior without a particle beam. The matched condition, in contrast, simulates a perfectly matched beam. In reality, the response at the beam pipe wall will most often lie between these extremes, with the particle beam absorbing some energy but not all. However, both conditions provide valuable insights for accelerator design. The matched condition corresponds to ideal power transmission of the field into the beam pipe. This corresponds to the information required for the on‐wall excitation used to predict particle motion in beam physics simulations. The infinite impedance condition, however, helps define the waveguide behavior under extreme conditions, where a firing of the pulse without a particle beam causes a full reflection of the field within the waveguide. The material selection and breakdown thresholds must take into account the resulting higher field intensity.

At an infinite impedance boundary, transmission line theory predicts a doubling of the voltage since the wave is fully reflected at the boundary and doubles back on itself. For all waveguides, the relative enhancement increased from 1 at low frequencies to 2 at higher frequencies (Figure 5). The reduced enhancement at lower frequencies, where wavelengths exceed the structure size, aligns with the diminishing relevance of transmission line theory in this regime. The maximum field enhancement and its frequency dependence can help select materials capable of withstanding the required voltages. Additionally, the fact that the boundary conditions themselves can contribute to frequency‐dependent effects should be considered in future simulations or experiments where alternative matching conditions at the inner radius are of interest.

In the pursuit of realizing a DWA, significant work remains to be done, particularly in defining the overall design through consideration of many interconnected factors. Achieving overall device optimization requires a comprehensive approach, taking into account factors including electronic capabilities, electric field generation, beam physics, treatment requirements, and material costs, among others. Research into these key areas is underway within our group, with notable advancements in testing high‐voltage solid‐state switches,^13^ the development of an open source beam physics model for DWAs,^14^ and the characterization of radial waveguide presented in this work. The enhancements observed in radial waveguides advance our objective in achieving high field gradients within the DWA, while our understanding of the dispersive effects observed at lower frequencies will inform requirements for future iterations of circuit designs. The development of the models presented here provides a vital advancement in our understanding of radial waveguide structures and provides a platform from which we can iteratively incorporate constraints and requirements from other aspects of the accelerator design.

## 5 CONCLUSIONS

Radial waveguides have been noted in the literature as having several advantages for their use as electric field generators in a DWA.^8^ Specifically, that they don't interfere with adjacent modules and that they produce high accelerating gradients.^8^ However, radial waveguides haven't been implemented in prototypes due to challenges in accounting for their radially varying impedance and the electric field dispersion that occurs in these structures. In this work, we show that the electromagnetic dispersion incurred in radial waveguides can be understood and accounted for if appropriate upstream pulse shaping can be implemented. The analytic expression derived using electromagnetic theory provided a means of parameterizing the impact of geometric and material waveguide properties. Furthermore, the analytic expressions were used as a means of validating simulation results, which in turn open up opportunities for further exploration of radial waveguides under non‐ideal conditions. For a uniform excitation, radial waveguides were found to introduce a passive enhancement of the applied field. This ultimately serves to reduces the upstream voltage required to produce a given field at the beam pipe relative to strip‐like geometries, making radial parallel plate waveguides a point of interest for future DWA development.

## CONFLICT OF INTEREST STATEMENT

The authors declare no conflicts of interest.
